# The role of home-based records in the establishment of a continuum of care for mothers, newborns, and children in Indonesia

**DOI:** 10.3402/gha.v6i0.20429

**Published:** 2013-05-06

**Authors:** Keiko Osaki, Tomoko Hattori, Soewarta Kosen

**Affiliations:** 1 Human Development Department, Japan International Cooperation Agency, Tokyo, Japan; 2 Health and Development Service, Tokyo, Japan; 3 National Institute of Helath Research and Development, Ministry of Health, Jakarta, Indonesia

**Keywords:** continuum of care, maternal, newborn and child health, personal health record, Maternal and Child Health handbook, Indonesia

## Abstract

**Background:**

The provision of appropriate care along the continuum of maternal, newborn, and child health (MNCH) service delivery is a challenge in developing countries. To improve this, in the 1990s, Indonesia introduced the maternal and child health (MCH) handbook, as an integrated form of parallel home-based records.

**Objective:**

This study aimed to identify the roles of home-based records both before and after childbirth, especially in provinces where the MCH handbook (MCHHB) was extensively promoted, by examining their association with MNCH service uptake.

**Design:**

This was a cross-sectional study using nationally representative data sets, the Indonesia Demographic and Health Surveys (IDHSs) from 1997, 2002–2003, and 2007. The IDHS identifies respondents’ ownership of home-based records before and after childbirth. Multivariate logistic regression was used to examine associations between record ownership and service utilisation in national data and data from two provinces, West Sumatra and North Sulawesi, where ownership of pre- and post-natal records served as a proxy for MCHHB ownership.

**Results:**

Pre- and post-natal record ownership increased from 1997 to 2007. Provincial data from 2007 showed that handbook ownership was associated with having delivery assisted by trained personnel [adjusted odds ratio (aOR): 2.12, 95% confidence interval (CI): 1.05–4.25], receiving maternal care (aOR: 3.92, 95% CI: 2.35–6.52), completing 12 doses of child immunisation for seven diseases (aOR: 4.86, 95% CI: 2.37–9.95), and having immunisation before and after childbirth (aOR: 5.40, 95% CI: 2.28–12.76), whereas national data showed that service utilisation was associated with ownership of both records compared with owning a single record or none.

**Conclusion:**

Our results suggest that pre- and post-natal home-based record use may be effective for ensuring service utilisation. In addition, since the handbook is an efficient home-based record for use throughout children's life courses, it could be an effective tool for promoting the continuum of MNCH care in Indonesia.

The provision of appropriate care along the maternal, newborn, and child health (MNCH) continuum is a core strategy for achieving the fourth United Nation's Millennium Development Goal (MDG4) to reduce child mortality and the fifth MDG (MDG5) to improve maternal mortality (1, 2). In many countries, gaps remain in MNCH care for priority interventions, especially in family planning, care around childbirth, and case management of childhood illnesses (3, 4).

Indonesia has a population of 240 million, including approximately 22 million aged under 5 years and approximately 4.4 million childbirths annually. Although Indonesia's under-five mortality rate has decreased from 85 per 1,000 live births in 1990 to 35 in 2010, moving towards its MDG of 28 by 2015, there has been less improvement in the neonatal mortality rate, which accounts for 48% of under-five deaths (5). The maternal mortality ratio in Indonesia has decreased from 600 per 100,000 live births in 1990 to 220 in 2010, though this still exceeds the MDG5 level of 150 by 2015 (5).

Priority intervention coverage for MNCH has increased in Indonesia (6). The percentage of mothers receiving antenatal care (ANC), performed at least once by a trained health provider, increased from 76% in 1991 to 93% in 2007. Furthermore, the proportion of deliveries attended by trained birth attendants increased from 32% in 1991 to 79% in 2007. However, coverage decreases as mothers move along the continuum of care – post-natal care coverage was 70% in 2007 – indicating the necessity to create care continuity in the pregnancy, delivery, and post-partum periods. In addition, achieving equitable coverage of attended delivery is still a distant goal in Indonesia (7). Immunisation coverage to protect children from infectious diseases varies; among children aged 12–23 months, 83% received measles immunisation and 77% received three doses of diphtheria, pertussis, and tetanus (DPT) vaccine, while 79% of newborns in 2008 were protected from tetanus before birth. Semba et al. stated that children who did not receive these immunisations were at higher risk of morbidity and mortality, highlighting the need to satisfy these important child survival efforts (8).

The maternal and child health (MCH) handbook (9) has been receiving increased attention (10, 11). It has two parts: a home-based record and information, education, and communication material related to MNCH. It attempts to increase awareness of the necessity of MNCH services (12) and has several key features that facilitate care continuity. Mothers receive it during their first ANC visit and refer to it as needed both at health service appointments and at home. Health personnel record details of the services delivered in the handbook and give guidance during service provision to help clients understand its contents and encourage them to share the information with their families (13).

Like other countries, Indonesia has used various types of parallel records, which has made record-keeping difficult (10). These disparate records have been gradually integrated into the MCH handbook (MCHHB). The MCHHB has been utilised since 1993 and verified on different islands of Indonesia to account for the country's diversity through technical cooperation projects conducted by the Japan International Cooperation Agency (JICA) with the Indonesian Ministry of Health (MOH). In practice, the MOH stopped providing maternal cards (*KMS Ibu Hamil*) in 1997, while continuing to distribute the child growth monitoring and immunisation card (*KMS*) as a transitional measure. A ministerial decree in 2004 (14) established the 48-page MCHHB (15) as a platform for stakeholders to work together for MNCH, including initiatives for promoting birth preparedness and complication readiness (16, 17), expansion of the immunisation programme (18), and introduction of new child growth standards (19). Over the past decade, development partners, such as the JICA, United Nations Children's Fund, World Health Organization, and United States Agency for International Development, as well as major professional organisations in Indonesia, have been supporting use of the handbook (20–22). The MOH encouraged the printing and distribution of the handbook and provided a partial budget for districts and cities to do so through the deconcentration fund around 2006.

One multi-centred pre- and post-intervention study with a comparison group reported that the use of maternal home-based records increased maternal and newborn service use, including ANC, tetanus toxoid (TT) immunisation, and post-natal care (23). Regarding MCHHB use, cross-sectional studies have demonstrated its association with better knowledge of maternal health, better communication between family members and health personnel, and better knowledge of using maternal health services, including ANC, TT immunisation, family planning, and deliveries assisted by trained health personnel. However, to examine the extent to which handbook use has persuaded women to utilise the care continuum, especially before and after childbirth, quantitative analysis is still needed (24–26).

The preliminary results of the IDHS 2007 found increases in both child record ownership and completion of child immunisation for ages 12– to 23 months, compared with IDHS 2002–2003 results (36.8% vs. 30.7% and 58.6% vs. 51.5%, respectively). Osaki et al. speculated on the possible contribution of the MCHHB to these increases by allowing more accurate immunisation coverage measurement, besides increasing awareness for immunisation (27). However, as the final report of the IDHS 2007 (28) showed that child immunisation completion had increased over the IDHS 2002–2003 numbers among both child record owners (73.3% vs. 70.9%) and non-owners (50% vs. 42.9%), further exploration of the association between home-based record ownership and immunisation completion is warranted. Therefore, our study aimed to identify the roles of home-based records both before and after childbirth, especially in provinces where the MCHHB was extensively promoted, by examining their association with MNCH service uptake.

## Materials and methods

### Data

We used nationally representative cross-sectional data from the IDHS 1997, 2002–2003, and 2007 (28–30). The IDHS questionnaires included variables related to home-based record ownership during pregnancy and after childbirth – whether mothers were given an antenatal card or an MCHHB and whether they had a post-natal immunisation record (28). Respondents who had received any records, including the MCHHB, no matter whether they had showed the records to the enumerator, were counted as home-based record owners. Persons who never received records or no longer own records were counted as non-home-based record owners. Data collected for children under 2 years, especially those over 9 months, enabled us to evaluate when timely immunisation coverage was necessary.

We analysed both national and provincial data from West Sumatra and North Sulawesi, where intensive MCHHB use was promoted by the JICA-MOH project (1998–2003) (31). After pilot studies on the MCHHB in Central Java province, a project was designed to verify handbook use in Sumatra and Sulawesi. Before this project, assisted delivery in two provinces (69.3% in West Sumatra and 45.7% in North Sulawesi) was higher than that of the national data (43.2%), but still a wide disparity within provinces was assumed (88.1% of the urban and 39.2% of the rural population in Outer Java-Bali I, to which those provinces belonged) (29). In these provinces, respondents with pre- and post-natal records were more likely to be MCHHB owners. While not available in 1995–1997, enough handbooks were procured in 2000–2002 and 2005–2007 to cover 95.1% and 121.3% of expected pregnancy, respectively. In the national data, there was a mix of records including the MCHHB and differing record cards. In 2000–2002, printed handbooks covered only 32% of expected pregnancies whereas it covered 48.2% in 2005–2007.

As this study involved analysis of secondary data with no individual case identification codes, it was deemed exempt by the relevant ethics review board.

### Statistical analysis

We set four dependent variables. First, we considered whether delivery was assisted by trained health personnel (e.g. physicians, midwives, auxiliary nurses). As pregnant women received the handbook and health personnel used it for monitoring and communicating with them throughout ANC, we assumed handbook owners were more likely to choose trained health personnel as delivery assistants. Second, we assessed the level of coverage across the maternal care continuum, including attending more than four ANC appointments, having assisted delivery, and receiving post-partum care within 1 week of giving birth. Third, we examined the completion of 12 doses of child immunisation based on the government's national immunisation programme (i.e. Bacille Calmette-Guérin vaccination, measles vaccination, three doses each of DPT and hepatitis B vaccines and polio vaccines 1–4) (32). Finally, we observed pre- and post-natal immunisation, including two doses of TT immunisation during pregnancy, leading to full coverage of the above-mentioned recommended immunisation schedule.

The independent variables consisted of home-based record ownership and possible confounders, such as respondents’ age, years of education, current marital status, household wealth quintiles, number of children per household, and whether their residence was urban or rural. For analysis of variables regarding immunisation, the children's ages were included in the set of independent variables.

We conducted logistic regression for both univariate and multivariate analyses to obtain crude (cORs) and adjusted odds ratios (aORs), respectively, as well as 95% confidence intervals (95% CIs) and significance values. Backward (Wald) stepwise elimination procedures were conducted to include all variables showing an overall *p*<0.20 in the regression model (33). To address possible collinearity, when a Spearman's rank-order correlation coefficient (*r*) of 0.8 or more was detected between two independent variables, one variable was eliminated from the model. The model was evaluated using the Chi-square (*p*<0.05) and Hosmer–Lemeshow goodness-of-fit tests (*p*≥0.05), using SPSS Version 18.0 (SPSS Japan Inc., Tokyo, Japan).

The IDHSs may have demonstrated more accurate and higher reporting when records were available for direct observation, while respondents without records answered all questions from memory. To control for this bias, we tested robustness by comparing two groups: non-owners and those with both records but were unable to show these records to enumerators.

## Results

### Home-based record ownership

Table 1 shows the prevalence of home-based record ownership for both national and provincial data, across the three IDHS data sets. In the IDHS 2007 national data, among respondents with children under 2 years, 66% owned records both before and after childbirth, while in the provincial data, 76.4% of respondents owned both records after sampling weight was adjusted.

**Table 1:** Possession of home-based records for 0 to 23-month-old children in the IDHSs from 1997, 2002–2003, and 2007^a^

	IDHS		
	
Possession of records^b^	1997 *n* (%)	2002–3 *n* (%)	2007 *n* (%)
National data			
Both records/MCHHB^c^	2,988 (50.3)	2,616 (52.9)	3,865 (66.0)
Child record only	1,599 (26.9)	1,221 (24.7)	986 (16.9)
Maternal record only	486 (8.2)	336 (6.8)	346 (5.9)
No record	864 (14.5)	772 (15.6)	657 (11.2)
Provincial data^d^			
Both records/MCHHB	99 (44.4)	115 (59.6)	161 (76.4)
Child record only	84 (37.6)	41 (21.3)	26 (12.5)
Maternal record only	10 (4.5)	18 (9.1)	6 (2.6)
No record	30 (13.5)	19 (10.1)	18 (8.5)

a Sampling weight-adjusted.

b Possession refers to stated ownership.

c MCHHB refers to ownership of both records in the provincial data, as we are assuming that having both records in provinces where MCHHB use was promoted is equivalent to having the MCHHB.

d Provinces refer to West Sumatra and North Sulawesi.

### Respondent characteristics

Table 2 shows respondents’ profiles, including owners of both records and those with a single record or none, in the IDHS 2007. The national data showed significant differences between the two groups in various socioeconomic indicators, except for the respondents’ age and marital status. Mothers with both records had longer education, older children and fewer children compared with those who did not have both. More mothers from households in higher wealth quintiles owned both records than those in lower wealth quintiles, and more mothers from households in urban areas owned both records than those in rural areas. The provincial data differed from the national, showing no significant effect of socioeconomic variables between the two groups, except for mother's education. We computed Spearman's correlations for each pair of variables and found that all were within acceptable limits (*r*_*s*_<0.53); therefore, we decided to employ all variables as independent variables for logistic regression (data not shown).

**Table 2:** Possession of home-based records and socioeconomic characteristics of respondents with 0 to 23-month-old children in the IDHS 2007

National data			Provincial data		
	
	Both records^a^ *n*=3,971	Single/no record *n*=2,355		MCHHB^b^ *n*=301	Single/no record *n*=96
Current respondent's age					
Mean (years)	28.36±6.01	28.11±6.38	Mean (years)	28.89±6.22	29.54±6.83
Respondent's education***			Respondent's education*		
Mean (years)	9.48±4.37	8.07±4.16	Mean (years)	9.78±3.53	8.93±3.87
Current marital status					
Married	3,904 (62.8)	2,317 (37.2)	Married	296 (75.5)	96 (24.5)
Ever married	67 (63.8)	38 (36.2)	Ever married	5 (100)	0 (0)
Parity***			Parity		
Primiparous	1,457 (66.8)	724 (33.2)	Primiparous	92 (76.7)	28 (23.3)
Multiparous	2,514 (60.7)	1,631 (39.3)	Multiparous	209 (75.5)	68 (24.5)
Current child's age**			Current child's age		
0**–**11 months	1,991 (59.8)	1,339 (40.2)	0**–**11 months	157 (78.1)	44 (21.9)
12**–**23 months	1,980 (66.1)	1,016 (33.9)	12**–**23 months	144 (73.5)	52 (26.5)
Wealth quintiles of the household***			Wealth quintiles of the household		
Poorest	778 (48.7)	820 (51.3)	Poorest	66 (77.6)	19 (22.4)
Poorer	749 (59.9)	501 (40.1)	Poorer	84 (74.3)	29 (25.7)
Middle	774 (65.4)	409 (34.6)	Middle	54 (71.1)	22 (28.9)
Richer	829 (71.3)	334 (28.7)	Richer	60 (78.9)	16 (21.1)
Richest	841 (74.3)	291 (25.7)	Richest	37 (78.7)	10 (21.3)
Residence***			Residence		
Rural	2,151 (57.8)	1,571 (42.2)	Rural	209 (78.3)	58 (21.7)
Urban	1,820 (69.9)	784 (30.1)	Urban	92 (70.8)	38 (29.2)

* *p*<0.05

** *p*<0.01

*** *p*<0.001.

Chi-square tests and *t*-tests were used for the analysis of categorical and continuous data, respectively. Numbers in parentheses are percentages and absolute numbers are total cases.

a Both records refer to both pre- and post-natal record ownership.

b MCHHB refers to ownership of both records in the provincial data, as we are assuming that having both records in provinces where MCHHB use was promoted is equivalent to having the MCHHB.

### Services at childbirth

In the national data, 83.6% of those with both records and 67.1% of those without both records delivered their babies with the assistance of trained health personnel. Univariate analysis showed a significant relationship between having deliveries assisted by trained health personnel and ownership of both records. Multivariate analysis indicated that ownership of both records was a significant predictor of assisted delivery (aOR: 1.71, 95% CI: 1.50–1.96), even when possible confounding factors regarding the respondent (e.g. age, parity, and education) and the household (e.g. wealth quintiles and urban or rural residence) were included in the regression equation (Table 3). In the provincial data, 88.7% of respondents who had both records and 80.2% of those who did not have both records had assisted delivery. The aOR for having assisted delivery was 2.1 times higher (95% CI: 1.05–4.25) among owners of both records than among those with no record or a single record (Table 4).

**Table 3:** Service uptake and possession of records in national data of the IDHS 2007

	Attendance at birth^a^			Maternal care continuity^a^			Completion of child immunisation with 12 doses for seven diseases^b^			Completion of child immunisation with two doses of TT b		
				
	*n* (%)	cOR (95% CI)	aOR (95% CI)^c^	*n* (%)	cOR (95% CI)	aOR (95% CI)^d^	*n* (%)	cOR (95% CI)	aOR (95% CI)^e^	*n* (%)	cOR (95% CI)	aOR (95% CI)^f^
Record ownership												
Both records^g^	3,319 (83.6)	2.50***(2.21–2.82)	1.71***(1.50–1.96)	2,610 (65.7)	2.62*** (2.36–2.91)	2.09*** (1.87–2.34)	1,190 (47.0)	2.65*** (2.28–3.07)	2.28*** (1.96–2.66)	742 (29.2)	2.42*** (2.02–2.88)	2.16*** (1.80–2.59)
Single/no record	1,580 (67.1)	Ref	Ref	995 (42.3)	Ref	Ref	324 (25.1)	Ref	Ref	189 (14.6)	Ref	Ref
Respondent's age (years)												
<20	234 (66.9)	Ref	Ref	155 (44.3)	Ref	Ref	53 (31.2)	Ref		29 (17.1)	Ref	
20–29	2,652 (77.6)	1.72*** (1.36–2.18)	1.25 (0.94–1.65)	1,941 (56.8)	1.65*** (1.33–2.07)	1.30* (1.02–1.67)	833 (41.0)	1.53* (1.10–2.15)		506 (24.9)	1.61* (1.07–2.43)	
30–39	1,808 (79.3)	1.90*** (1.49–2.43)	1.54** (1.13–2.10)	1,370 (60.1)	1.90*** (1.51–2.38)	1.65*** (1.26–2.16)	564 (39.1)	1.42* (1.01–1.99)		354 (24.5)	1.58* (1.04–2.39)	
40+	205 (73.2)	1.36 (.96–1.92)	1.50 (0.99–2.78)	139 (49.6)	1.24 (0.91–1.70)	1.34 (0.93–1.92)	64 (35.6)	1.22 (0.78–1.90)		42 (23.3)	1.48 (0.87–2.51)	
Respondent's education (years)												
<6	414 (52.8)	Ref	Ref	271 (34.6)	Ref	Ref	121 (25.2)	Ref	Ref	72 (14.9)	Ref	Ref
≥6, <9	1,186 (65.8)	1.72*** (1.45–2.04)	1.39** (1.15–1.67)	813 (45.1)	1.56*** (1.31–1.85)	1.29** (1.07–1.55)	371 (32.9)	1.46** (1.15–1.86)	1.27 (0.99–1.64)	227 (20.1)	1.44* (1.07–1.92)	1.30 (0.97–1.75)
9–11	1,090 (79.5)	3.47*** (2.86–4.20)	2.11*** (1.71–2.60)	806 (58.8)	2.70*** (2.25–3.24)	1.84*** (1.51–2.44)	329 (42.1)	2.16*** (1.68–2.78)	1.67*** (1.28–2.17)	204 (26.1)	2.01*** (1.49–2.70)	1.68** (1.24–2.29)
12+	2,203 (93.2)	12.31*** (9.94–15.23)	3.96*** (3.12–5.02)	1,712 (72.5)	4.98*** (4.19–5.92)	2.45*** (2.01–2.98)	692 (48.3)	2.78** (2.21–3.50)	1.83*** (1.42–2.36)	428 (29.9)	2.43*** (1.84–3.20)	1.97*** (1.46–2.66)
Child's age (months)	1.05*** (1.04–1.07)	1.06*** (1.04–1.08)		1.04*** (1.02–1.05)	1.04*** (1.02–1.06)							
Wealth quintile												
Poorest	844 (52.8)	Ref	Ref	537 (33.6)	Ref	Ref	262 (27.2)	Ref	Ref	174 (18.0)	Ref	Ref
Poorer	891 (71.3)	2.22*** (1.90–2.59)	1.70*** (1.44–2.00)	678 (54.2)	2.34*** (2.01–2.73)	1.96*** (1.68–2.30)	268 (35.2)	1.46*** (1.19–1.79)	1.21 (0.97–1.49)	173 (22.6)	1.33* (1.05–1.69)	1.10 (0.86–1.40)
Middle	1,000 (84.5)	4.88*** (4.05–5.88)	2.77*** (2.27–3.39)	746 (63.1)	3.37*** (2.88–3.95)	2.39*** (2.02–2.82)	283 (41.6)	1.91*** (1.55–2.35)	1.46** (1.17–1.82)	180 (26.4)	1.64*** (1.29–2.07)	1.23 (0.96–1.57)
Richer	1,058 (91.0)	9.00*** (7.20–11.25)	4.12*** (3.23–5.27)	787 (67.7)	4.14*** (3.52–4.86)	2.62*** (2.20–3.12)	362 (49.5)	2.63*** (2.14–3.22)	1.85*** (1.49–2.31)	223 (30.4)	1.99*** (1.59–2.50)	1.36* (1.06–1.74)
Richest	1,106 (97.7)	38.00*** (25.45–56.75)	11.82*** (7.70–18.15)	857 (75.7)	6.16*** (5.19–7.31)	3.28*** (2.70–3.98)	339 (49.4)	2.62*** (2.13–3.22)	1.72*** (1.36–2.18)	181 (26.4)	1.63*** (1.29–2.07)	1.02 (0.78–1.34)
Residential area												
Rural	2,534 (68.1)	Ref	Ref	1,876 (50.4)	Ref		777 (34.8)	Ref		509 (22.8)	Ref	
Urban	2,365 (90.8)	4.64*** (3.99–5.39)	1.67*** (1.40–1.99)	1,729 (66.4)	1.94*** (1.75–2.16)		737 (46.3)	1.61*** (1.42–1.84)		422 (26.5)	1.22** (1.05–1.42)	
Parity												
Primiparous	1,823 (83.6)	Ref	Ref	1,369 (62.8)	Ref	Ref	572 (43.2)	Ref		333 (25.1)		
Multiparous	3,076 (74.2)	0.57*** (0.50–0.65)	0.64*** (0.53–0.76)	2,236 (53.9)	0.70*** (0.63–0.77)	0.74*** (0.65–0.85)	942 (37.7)	0.80** (0.70–0.91)		598 (23.9)		
Current marital status												
Married	4,824 (77.5)	1.38 (0.90–2.12)		3,548 (57.0)			1,489 (39.7)	0.72 (0.44–1.16)	1.43 (0.87–2.36)	919 (24.5)	0.56 (0.30–1.04)	0.55 (0.29–1.03)
Widow/divorced	75 (71.4)	Ref		57 (54.3)			25 (32.1)	Ref	Ref	12 (15.4)	Ref	Ref

* *p*<0.05

** *p*<0.01

*** *p*<0.001.

a For 0- to 23-month-old children.

b For 9- to 23-month-old children.

c Chi-square test *p<*0.001; Hosmer–Lemeshow goodness-of-fit *p*=0.956.

d Chi-square test *p<*0.001; Hosmer–Lemeshow goodness-of-fit *p*=0.308.

e Chi-square test *p<*0.001; Hosmer–Lemeshow goodness-of-fit *p*=0.617.

f Chi-square test *p<*0.001; Hosmer–Lemeshow goodness-of-fit *p*=0.292.

g Both records refer to both pre- and post-natal record ownership.

**Table 4:** Service uptake and possession of records in provincial data of the IDHS 2007

	Attendance at birth^a^			Maternal care continuity^a^			Completion of child immunisation with 12 doses for seven diseases^b^			Completion of child immunisation with two doses of TT^b^		
				
	*n* (%)	cOR (95% CI)	aOR (95% CI)^c^	*n* (%)	cOR (95% CI)	aOR (95% CI)^d^	*n* (%)	cOR (95% CI)	aOR (95% CI)^e^	*n* (%)	cOR (95% CI)	aOR (95% CI)^f^
Record ownership												
MCHHB^g^	267 (88.7)	1.94* (1.05–3.59)	2.12* (1.05–4.25)	206 (68.4)	3.46*** (2.15–5.57)	3.92*** (2.35–6.52)	100 (53.8)	5.04*** (2.53-10.05)	4.86*** (2.37–9.95)	78 (41.9)	5.88*** (2.55–13.59)	5.40*** (2.28–12.76)
Single/no record	77 (80.2)	Ref	Ref	37 (38.5)	Ref	Ref	12 (18.8)	Ref	Ref	7 (10.9)	Ref	Ref
Respondent's age (years)												
<20	15 (75.0)			8 (40.0)	Ref	Ref	5 (45.5)			4 (36.4)		
20–29	169 (85.4)			112 (56.6)	1.95 (0.77–4.99)	1.49 (0.55–4.03)	52 (43.7)			42 (35.3)		
30–39	139 (90.3)			106 (68.8)	3.31* (1.27–8.63)	2.65 (0.95–7.40)	46 (44.7)			31 (30.1)		
40+	21 (84.0)			17 (68.0)	3.19 (0.93–10.88)	2.14 (0.58–7.90)	9 (52.9)			8 (47.1)		
Respondent's education (years)												
<6	31 (64.6)	Ref	Ref	23 (47.9)	Ref		9 (31.0)	Ref	Ref	7 (24.1)	Ref	Ref
6–8	67 (78.8)	2.04 (0.93–4.49)	1.50 (0.65–3.44)	42 (49.4)	1.06 (0.52–2.16)		16 (29.6)	0.94 (0.35–2.49)	0.76 (0.27–2.15)	11 (20.4)	0.80 (0.27–2.36)	0.73 (0.23–2.29)
9–11	75 (91.5)	5.88*** (2.22–15.57)	3.85* (1.39–10.68)	49 (59.8)	1.61 (0.79–3.31)		22 (42.3)	1.63 (0.62–4.26)	1.24 (0.44–3.47)	18 (34.6)	1.66 (0.60–4.64)	1.48 (0.49–4.47)
12+	171 (94.0)	8.53*** (3.65–19.93)	2.64* (1.04–6.67)	129 (70.9)	2.65** (1.38–5.07)		65 (56.5)	2.89* (1.21–6.89)	2.12 (0.84–5.39)	49 (42.6)	2.33 (0.92–5.90)	2.11 (0.75–5.87)
Child's age (months)		1.11** (1.04–1.18)	1.11** (1.04–1.18)		1.09** (1.02–1.16)	1.09* (1.02–1.16)						
Wealth quintile												
Poorest	62 (72.9)	Ref	Ref	38 (44.7)	Ref	Ref	20 (34.5)			18 (31.0)		
Poorer	91 (80.5)	1.53 (0.79–2.99)	1.37 (0.67–2.78)	67 (59.3)	1.80* (1.02–3.18)	1.92* (1.06–3.48)	29 (41.4)			24 (34.3)		
Middle	69 (90.8)	3.66** (1.47–9.11)	2.17 (0.82–5.76)	42 (55.3)	1.53 (0.82–2.85)	1.69 (0.88–3.26)	20 (47.6)			14 (33.3)		
Richer	75 (98.7)	27.82** (3.65–211.88)	11.60* (1.42–94.92)	61 (80.3)	5.03*** (2.48–10.22)	5.15*** (2.45–10.84)	29 (54.7)			21 (39.6)		
Richest	47 (100)	Na	Na	35 (74.5)	3.61** (1.65–7.89)	3.31** (1.43–7.64)	14 (51.9)			8 (29.6)		
Residential area												
Rural	216 (80.9)	Ref	Ref	154 (57.7)	Ref		72 (43.1)			61 (31.5)		Ref
Urban	128 (98.5)	15.11*** (3.62–63.12)	6.69* (1.51–29.69)	89 (68.5)	1.59* (1.02–2.48)		40 (48.2)			24 (28.9)		0.56 (0.29–1.06)
Parity												
Primiparous	103 (85.8)			69 (57.5)			38 (52.8)	Ref		30 (41.7)	Ref	
Multiparous	241 (87.0)			174 (62.8)			74 (41.6)	0.64 (0.37–1.10)		55 (30.9)	0.63 (0.36–1.10)	
Current marital status												
Married	339 (86.5)			239 (61.0)			110 (44.8)			83 (33.9)		
Widow/divorced	5 (100)			4 (80.0)			2 (40.0)			2 (40)		

* *p*<0.05

** *p*<0.01

*** *p*<0.001.

a For 0- to 23-month-old children.

b For 9- to 23-month-old children.

c Chi-square test *p<*0.001; Hosmer–Lemeshow goodness-of-fit *p*=0.988.

d Chi-square test *p<*0.001; Hosmer–Lemeshow goodness-of-fit *p*=0.946.

e Chi-square test *p<*0.001; Hosmer–Lemeshow goodness-of-fit *p*=0.984.

f Chi-square test *p <*0.001; Hosmer–Lemeshow goodness-of-fit *p*=0.303.

g MCHHB refers to ownership of both records in the provincial data, as we are assuming that having both records in provinces where MCHHB use was promoted is equivalent to having the MCHHB.

### Maternal care continuum before and after childbirth

Owners of both records had a better continuum of maternal care. According to national data, a higher proportion of mothers who possessed both records received continuous maternal care before and after the childbirth than those who possessed a single record or no record (65.7% vs. 42.3%, respectively). Predictors of continuous maternal care were in possession of both records (aOR: 2.09, 95% CI: 1.87–2.34), respondent's age, years of education, household wealth quintiles, and parity (Table 3). From the provincial data, uptake of a sequence of maternal care was associated with possession of both records (68.4% vs. 38.5%, respectively). Possession of both records remained as a predictor of continuous maternal care (aOR: 3.92, 95% CI: 2.35–6.52), together with respondent's age and household wealth quintiles in the regression equation (Table 4).

### Child immunisation completion

National data showed that among respondents with a child who was 9–23 months old, 1,190 (47%) respondents with both records and 324 (25.1%) respondents without both records completed 12 doses of immunisation. Furthermore, possession of both records (aOR: 2.28, 95% CI: 1.96–2.66), respondent's years of education, child's age, household wealth quintiles, and marital status were predictors of completing child immunisation (Table 3). Provincial data showed that 100 (53.8%) respondents with both records and 12 (18.8%) of those without both records completed immunisation. Possession of both records remained as a predictor of completing child immunisation (aOR: 4.86, 95% CI: 2.37–9.95), after including respondents’ years of education and child's age in the regression equation (Table 4).

### Immunisation before and after childbirth

Owners of both records were more likely to have children who were immunised against infectious diseases starting before birth and continuing until the recommended schedule was completed. The national data showed that 742 (29.2%) of the respondents who owned both records and 189 (14.6%) of those who did not have records had immunised their children against eight infectious diseases, including tetanus. Table 3 shows that ownership of both records, respondent's years of education, child's age, household wealth quintiles, and marital status were predictors of the outcome (aOR: 2.16, 95% CI: 1.80–2.59). The provincial data showed that 78 (41.9%) of the respondents who owned both records and 7 (10.9%) of those who did not have records had immunised children before and after birth. Predictors of the continuum of immunisation included record possession (aOR: 5.40, 95% CI: 2.28–12.76), respondent's years of education, child's age and urban versus rural residence (Table 4).

### Robustness test

In the data from respondent recall, owners of both records were more likely to have received most of the necessary services in both the national and the provincial data. Although assisted delivery was not significantly associated with record ownership in provincial data, the analysis still showed basically the same trends. This result suggests that bias from recall errors was minimal (Appendix 1).

## Discussion

### MCHHB use in Indonesia

The IDHS data showed the distribution of home-based record use in Indonesia. Provincial data indicated increased ownership of the MCHHB, as it is the most common form of record in West Sumatra and North Sulawesi. Because the handbook was distributed to pregnant women, it took several years to penetrate into respondents with children who were 0–23 months old. Furthermore, because the JICA-MOH project promoted MCHHB use starting with public health services and later expanding to private services, the increase in service points may have contributed to the increase in handbook ownership in recent years.

Provincial data suggested that socioeconomic variables have less of an influence on handbook ownership compared with national data. Several efforts to promote the handbook might have contributed to its equitable distribution. For example, contests and seminars on the handbook were organised for community health vounteers and religious leaders; mothers’ classes were introduced to provide mothers with health education using the handbook; and midwifery and nursing academies were approached to introduce the handbook into their curriculum, so that future health providers, particularly those working closely with mothers at the community level, would be familiar with the handbook (34). The high record ownership for younger children observed in the provincial data may indicate that younger children had more opportunities to obtain their record if it was provided to their mothers before childbirth, such as in the form of the MCHHB. Providing the MCHHB before birth may have addressed mothers’ need for information about basic infant and child development and care, which is often lacking in developing countries (35). However, further investigation would be needed to determine whether MCHHB distribution increases earlier service access after childbirth.

### Home-based record ownership and health outcomes

Bhutta et al. identified three priority coverage gaps – in family planning services, skilled care during and after childbirth, and clinical care for sick children – that health care interventions must target to achieve MDGs 4 and 5 (4). Our study, which showed an association between possession of pre- and post-natal records and use of maternal care services, addressed the second of these gaps.

Our results appear to confirm and extend those of previous studies showing that home-based record use is associated with better utilisation of maternity care and immunisations. Our study shows that possession of both records (before and after childbirth) is associated with a better maternity care continuity and more complete immunisation than having only one record or none. The results using data from two provinces with a population of 7.11 million (36) support the feasibility of scaling up the distribution of the MCHHB as a tool for improving the national health system.

Regarding the two child immunisation variables, we observed associations between completion of child immunisation before and after childbirth and possession of both records. The provincial data for possession of records give us some indication of the direction of causation in these relationships. The MCHHB, the most common form of record in these provinces, are intended for distribution to pregnant women at ANC but not to children during immunisation. Instead, if these children have no other form of records, child cards are often distributed to them when they are immunised (28). Thus, it is unlikely that getting children immunised led to handbook ownership, and it is instead likely that acquiring the handbook led to the immunisation of children.

Increases in service demand and efficiency in service supply may have resulted in the increased coverage of the studied services (24, 26). In addition, equitable distribution of home-based records across wealth quintiles, which we observed in the provincial data, may have resulted in enhanced equity in service access. Wulansari described that one of the responses by community members in the Village Health Preparedness Programme (*Program Kesehatan Desa Siaga*), a national community empowerment programme, was to use the MCHHB to monitor and bring children to immunisation programmmes (37). If equitable tools are available in the community, they can be used to facilitate equitable access to essential services to achieve MDGs 4 and 5. Nevertheless, record ownership both before and after childbirth appears to be associated with service uptake. As the MCHHB enables the complete and efficient retention of MNCH records before and after childbirth, it can be an effective tool for promoting care continuity.

### Limitations

We used ownership of home-based records as a proxy for MCHHB ownership in the provincial data, but the possibility remains that respondents in these provinces held two separate record cards. Owning two separate cards indicates that women were obtaining two services, and as such, they would have higher utilisation than women obtaining only one service. Moreover, because provinces in this analysis might have had better services since before MCHHB use compared with national coverage, then the associations observed would be limited to settings where a certain level of service was available. Therefore, it would still be worthwhile to evaluate data that covers a wider part of the country, especially using data that identifies handbook ownership directly in order to confirm the assumptions made in this analysis.

Our cross-sectional study design limited our inference of causal relationships between home-based record ownership and service uptake. Thus, we cannot conclude that these observed relationships were only due to record ownership. These relationships could also reflect better access to or better services provided by clinics that provide records to their clients, or unobserved factors among clients who keep good records on their health and the health of their children. This analysis identified the overall or total-derivative effect of providing home-based records on each outcome, encompassing the availability and accessibility of services and the responses of mothers and health personnel in their optimal or partial use (24). However, as Duflo et al. explained, the total-derivative effect is still of interest to policymakers, because it shows what will happen to outcome measures after an input is provided and agents are reoptimised, presenting the ‘real’ effect of the policy on outcomes of interest (38). Our analysis provides a basis for further studies to explore the effectiveness of the MCHHB in creating MNCH care continuity.

## Conclusion

As the MCHHB is an efficient means of providing home-based records before and after childbirth throughout children's life courses, it could be an effective tool for promoting the continuum of MNCH care. Despite their limited resources, the government and its development partners have important roles in providing an efficient continuum of MNCH care by integrating services. The theory of service integration needs to be transformed into practice. The MCHHB is a potentially useful tool for integrating services for mothers and children by putting mothers and children at the centre of the country's health system.

## Authors’ contributions

K. O. contributed to the study concept and design. K. O. and S. K. analysed and interpreted the data. T. H. collected and summarised information on the implementation of the MCH handbook in Indonesia. K. O. drafted this article and revised it, based on critical reading and comments from all the authors. All of the authors read and approved this final manuscript. K. O. is the guarantor of this article.
